# A Fluorine-19 Magnetic Resonance Probe, Shiga-Y5, Downregulates Thioredoxin-Interacting Protein Expression in the Brain of a Mouse Model of Alzheimer’s Disease

**DOI:** 10.3390/molecules26175342

**Published:** 2021-09-02

**Authors:** Aslina Pahrudin Arrozi, Zulzikry Hafiz Abu Bakar, Hiroyasu Taguchi, Daijiro Yanagisawa, Ikuo Tooyama

**Affiliations:** Molecular Neuroscience Research Center, Shiga University of Medical Science, Seta Tsukinowa-cho, Otsu 520-2192, Japan; aslina@belle.shiga-med.ac.jp (A.P.A.); zikry@belle.shiga-med.ac.jp (Z.H.A.B.); taguti@belle.shiga-med.ac.jp (H.T.)

**Keywords:** curcumin, oxidative stress, thioredoxin, *TXNIP*, Alzheimer’s disease

## Abstract

Thioredoxin-interacting protein (*TXNIP*) is involved in multiple disease-associated functions related to oxidative stress, especially by inhibiting the anti-oxidant- and thiol-reducing activity of thioredoxin (*TXN*). Shiga-Y5 (SY5), a fluorine-19 magnetic resonance probe for detecting amyloid-β deposition in the brain, previously showed therapeutic effects in a mouse model of Alzheimer’s disease; however, the mechanism of action of SY5 remains unclear. SY5 passes the blood–brain barrier and then undergoes hydrolysis to produce a derivative, Shiga-Y6 (SY6), which is a *TXNIP*-negative regulator. Therefore, this study investigates the therapeutic role of SY5 as the prodrug of SY6 in the thioredoxin system in the brain of a mouse model of Alzheimer’s disease. The intraperitoneal injection of SY5 significantly inhibited *TXNIP* mRNA (*p* = 0.0072) and protein expression (*p* = 0.0143) induced in the brain of APP/PS1 mice. In contrast, the levels of *TXN* mRNA (*p* = 0.0285) and protein (*p* = 0.0039) in the brain of APP/PS1 mice were increased after the injection of SY5. The ratio of *TXN* to *TXNIP*, which was decreased (*p* = 0.0131) in the brain of APP/PS1 mice, was significantly increased (*p* = 0.0072) after the injection of SY5. These results suggest that SY5 acts as a prodrug of SY6 in targeting the thioredoxin system and could be a potential therapeutic compound in oxidative stress-related diseases in the brain.

## 1. Introduction

Thioredoxin-interacting protein (*TXNIP*) plays an important role in the redox system, but it is implicated differently in many diseases, including cancers, diabetes, and neurodegenerative disorders [1]. Additionally, *TXNIP* is an endogenous intracellular inhibitor of thioredoxin (*TXN*), an anti-oxidant protein in the thiol-reductase redox system [2]. *TXNIP* expression is highly triggered by glucose and is increased in the beta-cells (β-cells) of patients with diabetes [3], inhibiting *TXN* function and inducing increased levels of freely diffusible molecular hydrogen peroxide; it contributes to oxidative stress and eventually β-cell death [4]. Studies showed that *TXNIP* inhibition prevented β-cell apoptosis and protected against diabetes [5,6]. In contrast, *TXNIP* was underexpressed in most cancers, leading to high cell proliferation [7]. Mechanisms of *TXNIP* downregulation include the interaction of microRNAs with the 3′ untranslated region (UTR), the binding of transcription factors, and epigenetic changes in the promoter region of *TXNIP* [8]. Thus, inducing *TXNIP* expression could remarkably block the cell cycle and suppress tumor cells [9,10].

*TXNIP*’s involvement in Alzheimer’s disease (AD) is mostly associated with inflammation and accompanied by other processes, such as amyloid β (Aβ) deposition and neurofibrillary tangles [11,12]. Recent studies have demonstrated that *TXNIP* protein expression was upregulated and colocalized near Aβ plaques and phosphorylated tau (p-tau) [13]. In contrast, in a separate study, *TXN* protein levels were downregulated in the brains of AD mice [14,15]. A consistent finding showed that *TXNIP* protein expression increased significantly in the hippocampus and frontal cortex of Aβ precursor protein and presenilin 1 (APP/PS1) transgenic mice and primary cultured mouse cerebral cortex neurons HT22 mouse hippocampal cells treated with Aβ peptide [16]. *TXNIP* remained an exclusive marker in microglia, neurons, astrocytes, and endothelial cells [17,18,19]. *TXNIP* has been proposed as an essential mediator of nucleotide-binding oligomerization domain-, leucine-rich repeat- and pyrin domain-containing 3 (NLRP3) inflammasome activation and the eventual formation of activated caspase 1 [13]. Preventing the interaction of *TXNIP* with NLRP3 demonstrated positive effects by reversing or restraining AD pathology [20,21]. Considering the involvement of *TXNIP* in the pathology of the disease, targeting these proteins might be the possible therapeutic target for intervention.

**Figure 1:**
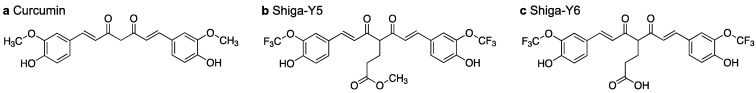
Chemical structure of curcumin, Shiga-Y5 and Shiga-Y6. (**a**) Curcumin, a yellow–orange pigment in turmeric. (**b**) Shiga-Y5 is a fluorinated curcumin derivative with methyl ester groups at the C-4 position. (**c**) Shiga-Y6 with carbonic acid groups at the C-4 position was obtained by the hydrolysis of Shiga-Y5.

Curcumin (1,7-bis(4-hydroxy-3-methoxyphenyl)-1,6-heptadiene-3,5-dione) (Figure 1a), a polyphenol also called diferuloylmethane, is the low molecular weight yellow–orange pigment derived from a rhizomatous herbaceous perennial plant (*Curcuma longa*) of the ginger family known as turmeric [22]. We previously reported a novel curcumin derivative, 1,7-Bis(4′-hydroxy-3′-trifluoromethoxyphenyl)-4-methoxycarbonylethyl-1,6-heptadiene-3,5-dione, named Shiga-Y5 (SY5) (Figure 1b), which was synthesized in our laboratory as a fluorine-19 magnetic resonance (^19^F MR) probe to detect amyloid deposition in the Tg2576 mouse [23]. SY5 contains six fluorine atoms and bears a substitution at the C-4 position, influencing the ratio of keto to enol tautomer and its effects on amyloid β (Aβ) aggregation [24]. Our previous study demonstrated that only SY5 is effective against Aβ toxicity; it improved cognitive impairment, inhibited amyloid deposition and reduced glial activation in APP/PS1 mice [25]. 

Several studies have demonstrated the therapeutic effect of curcumin, as seen by the increased *TXN* protein expression [26] and decreased *TXNIP* protein expression [27] in cells subjected to oxidative stress. A curcumin derivative with the carbonic acid group named Shiga-Y6 (SY6) (Figure 1c) showed negative regulation on *TXNIP* expression in culture cells exposed to high glucose and endoplasmic reticulum stress inducer [28]. Our preliminary study showed that SY5 passes the blood–brain barrier (BBB), and the methyl ester group undergoes hydrolysis in the brain to produce SY6; however, the mechanism remains unclear. This finding suggests that acting as a prodrug of SY6 is a possible mechanism of action of SY5 to inhibit *TXNIP*, leading to the therapeutic effect in the AD model mice. Here, we investigated the role of SY5 as the prodrug of SY6 in the thioredoxin system, which might be a possible target for intervention in AD. 

## 2. Results

### 2.1. Gene Expression of TXN and TXNIP

**Figure 2:**
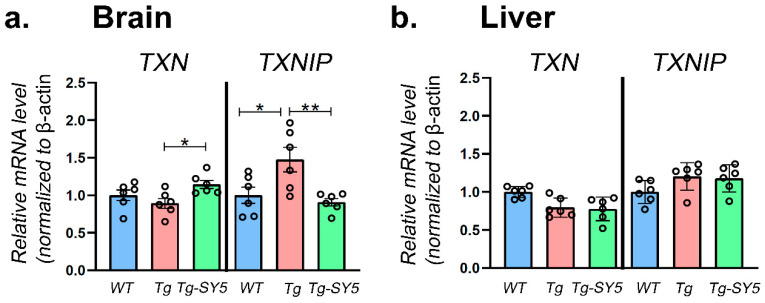
The *TXN* or *TXNIP* gene expression level in the amyloid precursor protein and presenilin 1 double transgenic (APP/PS1) mice in (**a**) brain and (**b**) liver. Data are presented as mean ± SEM (*n* = 6). Significance: ** *p* < 0.01, * *p* < 0.05.

Our previous study using SY5 indicated that the dose of 200 mg/kg was tolerated and can cross the BBB after intravenous injection in APP/PS1 mice [23]. In this study, the same dose of SY5 (200 mg/kg) was administered by intraperitoneal injection (i.p). After 2 h, the mice were sacrificed, the brain was quickly removed, and the cerebral cortex and liver were isolated and cut in half for the gene and protein expression analysis. In the brain’s cerebral cortex, the *TXN* mRNA level was unchanged in APP/PS1 mice compared with WT mice. However, with SY5 treatment, the *TXN* mRNA level was significantly increased (*p* = 0.0285). In contrast, *TXNIP* mRNA level was significantly increased (*p* = 0.0224) in APP/PS1 mice compared with WT mice, and with SY5 treatment, *TXNIP* mRNA level was significantly decreased (*p* = 0.0072) compared to without treatment in APP/PS1 mice (Figure 2a). There were no significant differences in the liver in *TXN* or *TXNIP* mRNA levels between APP/PS1 and WT mice and between APP/PS1 mice with or without SY5 treatment (Figure 2b).

### 2.2. Protein Expression of TXN and TXNIP

**Figure 3:**
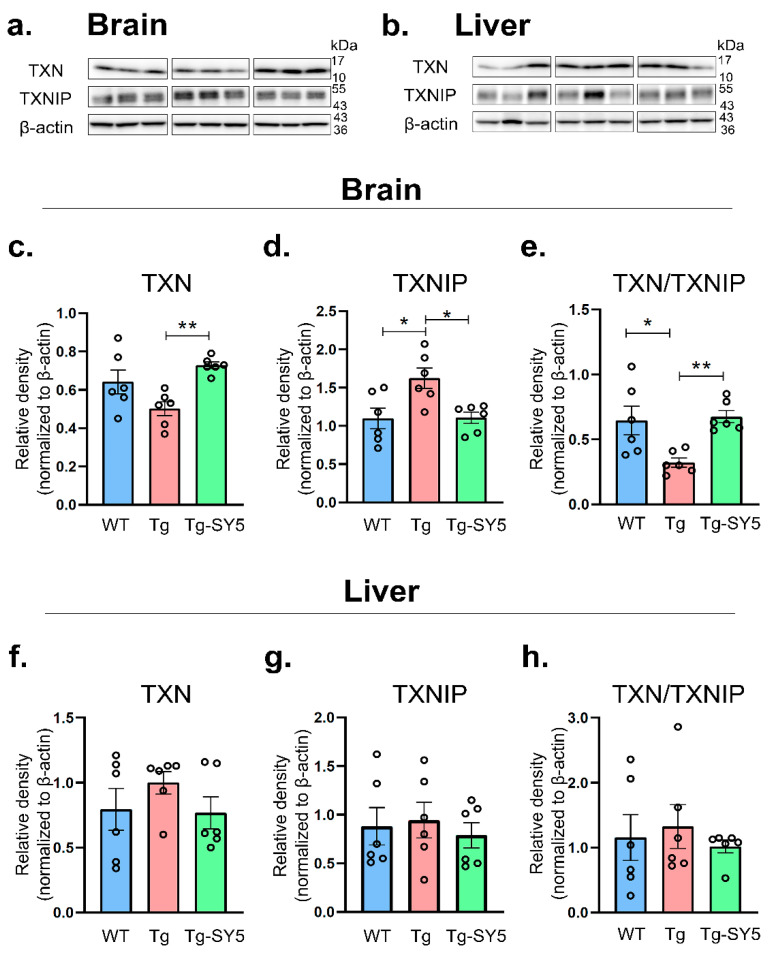
The *TXN* or *TXNIP* protein expression level in the APP/PS1 mice. Representative images of Western blotting for *TXN*, *TXNIP*, and β-actin in (**a**) the brain and (**b**) liver. Densitometric analysis of *TXN* or *TXNIP* and calculation of the ratio of *TXN*/*TXNIP* in the cerebral cortex of the brain (**c**–**e**) and the liver (**f**–**h**). Data are presented as mean ± SEM (*n* = 6). Significance: ** *p* < 0.01, * *p* < 0.05. Full-length blots are presented in Supplementary Figures S1 and S2.

We further investigated the effect of SY5 on the protein level of *TXN* and *TXNIP* in APP/PS1 mice. Protein extracts from the cerebral cortex and the liver were electrophoresed on SDS gel, and the protein bands of *TXN* and *TXNIP* were detected at the estimated size of 12 and 52 kDa, respectively (Figure 3a,b). Full-length blots are presented in Supplementary Figures S1 and S2. In the brain’s cerebral cortex, the *TXN* protein expression level showed a decreasing trend but was not statistically significant in APP/PS1 mice compared with WT mice. In contrast, there was a significant increase (*p* = 0.0039) in the *TXN* protein expression level with SY5 treatment than without treatment in APP/PS1 mice (Figure 3c). Furthermore, the *TXNIP* protein expression level demonstrated a significant increase (*p* = 0.0126) in APP/PS1 mice compared with WT mice; the increased level of *TXNIP* was significantly reversed (*p* = 0.0143) with SY5 treatment compared with without treatment in APP/PS1 mice (Figure 3d). Additionally, the ratio of *TXN*/*TXNIP* significantly decreased (*p* = 0.0131) in APP/PS1 compared with WT mice; however, the decreased ratio of *TXN*/*TXNIP* was more significantly reversed (*p* = 0.0072) with SY5 treatment than without treatment in APP/PS1 mice (Figure 3e). Meanwhile, in the liver, consistent with the mRNA level, there were no significant differences in *TXN* or *TXNIP* protein expression level and the ratio of *TXN*/*TXNIP* between APP/PS1 and WT mice and between APP/PS1 mice with or without SY5 treatment (Figure 3f–h).

## 3. Discussion

In this study, we investigated the role of SY5 in regulating *TXN* and *TXNIP* in APP/PS1 mice. It had been initially hypothesized that targeting *TXNIP* by lowering its levels would prevent the inhibitory binding to *TXN*, which might reduce oxidative stress. Our results show that the *TXNIP* protein expression level was initially upregulated in the brain of APP/PS1 mice; however, with SY5 treatment, it was downregulated and accompanied by the upregulation of *TXN* at the transcriptional and translational levels. In contrast, we found no changes in *TXN* or *TXNIP* levels in the liver, suggesting that SY5 acts as a signaling molecule targeting the thioredoxin system in the brain of APP/PS1 mice.

Consistent with the previous study, the protein expression of *TXNIP* increased significantly without changes in *TXN* in the brain of APP/PS1 mice at 9 and 12 months of age when Aβ deposits had developed compared with wild-type mice [16]. Several studies have demonstrated that *TXNIP* is required to activate NLRP3 inflammasome, a prevalent proposed mechanism in mediating inflammation in AD [13,29,30]. Activated NLRP3 recruited the adapter protein apoptosis-associated specklike (ASC) and pro-caspase 1, leading to caspase 1 production and subsequent interleukin-1β (IL-1β) maturation and release, activating signaling pathways and resulting in neuroinflammation and neuronal death [13,31,32]. Inhibiting *TXNIP* was elevated during disease development or in the presence of cellular stress factors, which showed several benefits, particularly in reversing the pathological outcomes. For example, the inhibitory effects of curcumin on *TXNIP* may have caused failed activation of the NLRP3 inflammasome, subsequently suppressing the upregulation of proinflammatory cytokines and improving paraquat-induced oxidative stress in lung fibroblast cells [27]. Similar effects of curcumin were also demonstrated whereby *TXNIP*/NLRP3 inflammasome activation was inhibited, suppressing endoplasmic reticulum stress and protecting neuronal cell survival in mice hippocampus-induced glutamate neurotoxicity [33].

Previously, we have synthesized and reported at least two ^19^F MR probes containing different novel contrast agents to detect amyloid deposits in the brain of transgenic mouse models of AD [34]. These include curcumin (Shiga-Y) [23] and styrylbenzoxazole derivatives (Shiga-X) [35], which possess fluorine substituents to detect ^19^F MR signals. MR sensitivity of ^19^F is high compared with various nuclei other than ^1^H (^1^H, 100%; ^19^F, 83%; ^31^P, 6.6%; ^13^C, 1.6%). No detectable fluorine atoms exist in biological tissues (fluorine in bones and teeth is inappropriate for ^19^F MRI relaxation time), which could result in low endogenous background noise. Furthermore, the ^19^F atom is a nonradioactive isotope comprising 100% of naturally abundant fluorine. Thus, ^19^F MRI is a highly sensitive, readily available, low-background, and cost-effective approach once a suitable high-quality probe has been developed. Among more than 40 ^19^F MR probes containing curcumin derivatives synthesized in our laboratory, the one typically used for amyloid imaging in the mouse brain using MRI was SY5 [36]. SY5 contained six ^19^F atoms in two CF_3_ groups, which display a single sharp ^19^F nuclear magnetic resonance (NMR) signal in solution and could bind to senile plaques in human brain sections. However, when these chemicals were intravenously injected into the tail veins of the APP transgenic mouse model (Tg2576), only SY5 passed through the BBB and bound to Aβ plaques (Supplementary Figure S3). In contrast, SY6 did not bind to Aβ plaques due to poor permeability into the brain (Supplementary Figure S3) [36].

Previous studies in our laboratory showed that SY6 demonstrated significantly greater activity in inhibiting *TXNIP* expression and enhancing *TXN* expression in cellular models of diabetes, endoplasmic reticulum stress, and inflammation while possessing the anti-inflammatory properties of native curcumin [28]. However, SY6 could not cross the BBB, limiting its further investigation in an animal model. Our preliminary study showed that SY5 could be converted to SY6 when entering the brain; however, the mechanism remains unclear. Thus, we speculate that the inhibitory effect of SY5 on *TXNIP* and TXN upregulation in the brain was possibly from the action exerted by SY6. However, the action exerted by SY5 on *TXNIP* may be independent of conversion to SY6. Further study is needed to discover more about the mechanism of action of SY5.

We also demonstrated that in the microenvironment surrounding Aβ plaques, SY5, which bears a substitution at the C-4 position, likely exists in equilibrium between the free (keto) and bound (enol) forms to influence their effects on amyloid β (Aβ) aggregation. Additionally, only the enol form of this compound can bind to Aβ aggregates/fibrils [24] and Aβ oligomers [37] but not to Aβ monomers. Furthermore, ^19^F NMR signals displayed by SY5 showed dose-dependently as it was hardly detected at a dose of 50 mg/kg but easily detected at a dose of 100 mg/kg and tolerable up to a dose of 200 mg/kg [23]. Histological analysis of SY5 following intravenous injection in APP/PS1 mice showed strong fluorescent signals in the cortex and hippocampus, with most of this signal colocalized with Aβ immunoreactivity [23]. Furthermore, we demonstrated that only SY5 inhibited behavioral deficit, as seen in the water maze test, a significant reduction in latencies for the platform at days 5 and 6, compared with day 1 in APP/PS1 mice besides comparable time spent in the targeted quadrant to that in WT mice [25]. Our previous studies show that Aβ in the form of fibrils and oligomers are targeted by SY5 and improved cognitive function in APP/PS1 mice. Possibly, there will be more targeted proteins to explain the mechanisms of action and therapeutic effects of this compound.

There were some limitations in this study, including no investigation regarding changes in *TXNIP* levels in SY5-injected wild-type mice, the effect of SY5 on cognitive function and Aβ accumulation in the brain, and the detailed mechanism of how SY5 acts as a *TXNIP* modulator in the brain. Further study is needed to clarify these points.

Summarily, these data indicate directions for future studies for characterizing the properties of curcumin derivatives. We have produced many chemically modified curcumin derivatives that can be tested in these simple assays to modulate *TXNIP* and *TXN* expression.

## 4. Materials and Methods

### 4.1. Animals

All animal experiments were conducted according to the National Institutes of Health Guide for the Care and Use of Laboratory Animals and approved by the Institutional Ethics Committee of Shiga University of Medical Science (2021-6-10). APP/PS1 mice with a C57BL/6 background (Jackson Laboratory, Bar Harbor, ME, USA), expressing a chimeric mouse/human amyloid precursor protein with the K594N and M595L mutations linked to Swedish familial AD (Mo/HuAPP695swe) and human PS1, carrying the exon 9 deletion associated with familial AD [38] were used in this study. The mice were housed in standard laboratory cages at 23 °C with free access to water and food in an SPF animal facility and maintained with a 12 h light/dark cycle with lights on from 8:00 a.m. to 8:00 p.m.

### 4.2. Treatment Groups

Shiga-Y5 was dissolved at a concentration of 10 mg/mL in saline containing 10% Cremophor EL. The WT mice (*n* = 6) aged 10–12 months received an intraperitoneal injection (i.p.) of normal saline containing 10% Cremophor EL. The APP/PS1 mice aged 10–12 months were divided into two groups, with six mice in each group and received i.p. normal saline (Tg) and Shiga-Y5 at 200 mg/kg (Tg-SY5). After 2 h, the mice were sacrificed under deep anesthesia using sodium pentobarbital (200 mg/kg, i.p.); the brain was quickly removed from each mouse. The cerebral cortex tissues from the right hemisphere and the liver were isolated and snap-frozen in liquid nitrogen and stored at −80 °C until further use for gene and protein expression analysis.

### 4.3. Preparation of Protein Extracts from Brain and Liver Tissue

Brain and liver tissue were homogenized in 10 volumes of Tris-buffered saline (TBS; 25 mM Tris-HCl (pH 7.5), 150 mM NaCl, 1 mM EGTA, protease inhibitor cocktail (Roche Diagnostic, Mannheim, Germany) and phosphatase inhibitor cocktail (Roche Diagnostic)) added to Triton X-100 (1% final concentration), kept on ice for 60 min and centrifuged at 10,000× *g*, 4 °C for 15 min. The supernatant was collected, and the protein concentration was determined using a Protein Assay Bicinchoninate kit (Nacalai Tesque, Kyoto, Japan).

### 4.4. Western Blotting

Proteins (10 µg) in 4x sample buffer and 10x reducing buffer (Life Technologies, Waltham, MA, USA) were denatured at 95 °C for 10 min, applied to the lanes of precast 15% polyacrylamide gels (Wako Pure Chemicals, Osaka, Japan), electrophoresed, and transferred to polyvinylidene difluoride membranes (Immobilon-P; Merck Millipore, MA, USA). The membranes were blocked with 5% skim milk in Tris-buffered saline containing 1% Tween 20 (TBST) at room temperature for 1 h, followed by overnight incubation at 4 °C with the primary antibodies including rabbit monoclonal antibodies against thioredoxin (ab273877; 1:1000; Abcam, Cambridge, UK), *TXNIP* (ab188865; 1:2000, Abcam) and mouse monoclonal antibody against β-actin (1:5000; Santa Cruz Biotechnology, Dallas, TX, USA). The membranes were further incubated with horseradish peroxidase-conjugated goat polyclonal antibody against rabbit immunoglobulin (Ig)G (1:10,000; Jackson ImmunoResearch, West Grove, PA, USA) and mouse IgG (1:10,000; Jackson ImmunoResearch) for 1 h at room temperature. Immunoreactivity proteins were visualized with a chemiluminescence substrate (SuperSignal West Pico PLUS Chemiluminescence Substrate; Thermo Fisher Scientific, Waltham, MA, USA) using Western blotting imager (Fusion Solo S, Vilber, Nantes, France). The band density was analyzed using image processing software (ImageJ; National Institutes of Health, Bathesda, MD, USA).

### 4.5. RNA Isolation and Quantitative Polymerase Chain Reaction (qPCR)

Total RNA was extracted from the cerebral cortex and the liver using RNeasy Lipid Tissue Mini and RNeasy Plus Universal Mini Kit (Qiagen, Hilden, Germany), respectively, following the manufacturer’s protocol. RNA samples were reverse transcribed into cDNA using ReverTra Ace qPCR RT Master Mix with gDNA Remover kit (Toyobo, Osaka, Japan) in accordance with the manufacturer’s protocol. qPCR was conducted in duplicate 10 µL reactions containing 2-µL cDNA, 5-µL TaqMan Fast Advanced Master Mix (Applied Biosystem, Waltham, MA, USA), 0.5-µL TaqMan Gene Expression assay (*TXN*, Mm00726847_s1; *TXNIP*, Mm00452393_m1; ACTB, Mm01205647_g1) (Applied Biosystem) and 3.5 µL of RNase free water on a LightCycler 480 instrument (Roche Diagnostic) using the following cycling parameters: Uracil N-glycosylase incubation at 50 °C for 2 min, polymerase activation at 95 °C for 2 min, followed by 40 cycles of denaturation at 95°C for 1 s, annealing/extension at 60 °C for 20 s.

### 4.6. Statistical Analysis

Statistical analyses were performed using GraphPad PRISM v.8 software (GraphPad Software, San Diego, CA, USA). Data are presented as the mean ± standard error of the mean (SEM). Statistical comparisons between groups were performed using one-way ANOVA followed by Sidak post hoc test. The statistical significance of all tests was set at *p* < 0.05.

## 5. Patents

Shiga University of Medical Science obtained a Japanese patent (JP2012-260046) on SY5 and SY6, with D.Y., H.T., and I.T. named as the inventors, and has submitted another Japanese patent application (JP2020-141272) on SY6, with D.Y., H.T., and I.T. named as the inventors.

## Data Availability

The data presented in this study are available on request from the corresponding author.

## Supplementary Materials

The following are available, Figure S1: Full-length blots in Figure 2a, Figure S2: Full-length blots in Figure 2b, Figure S3: ^19^F MRI using Shiga-Y5 and Shiga-Y6.

## Sample Availability

Sample of the compounds are available from the authors.
