# Supplementary material for: A Fluorine-19 Magnetic Resonance Probe, Shiga-Y5, Downregulates Thioredoxin-Interacting Protein Expression in the Brain of a Mouse Model of Alzheimer’s Disease
Source: Molecules. 2021 Sep 2;26(17):5342. doi: 10.3390/molecules26175342 (PMC8433978; doi:10.3390/molecules26175342)
Supplement: Supplementary file 1 [file molecules-26-05342-s001.zip › molecules-1340914 Supplementary Information.pdf]

Supplementary Information

A fluorine-19 MR probe, Shiga-Y5, downregulates thioredoxin-interacting protein expression in the brain of a mouse model of Alzheimer's disease

Aslina Pahrudin Arrozi, Zulzikry Hafiz Abu Bakar, Hiroyasu Taguchi, Daijiro Yanagisawa, Ikuo Tooyama

Supplementary Figure S1

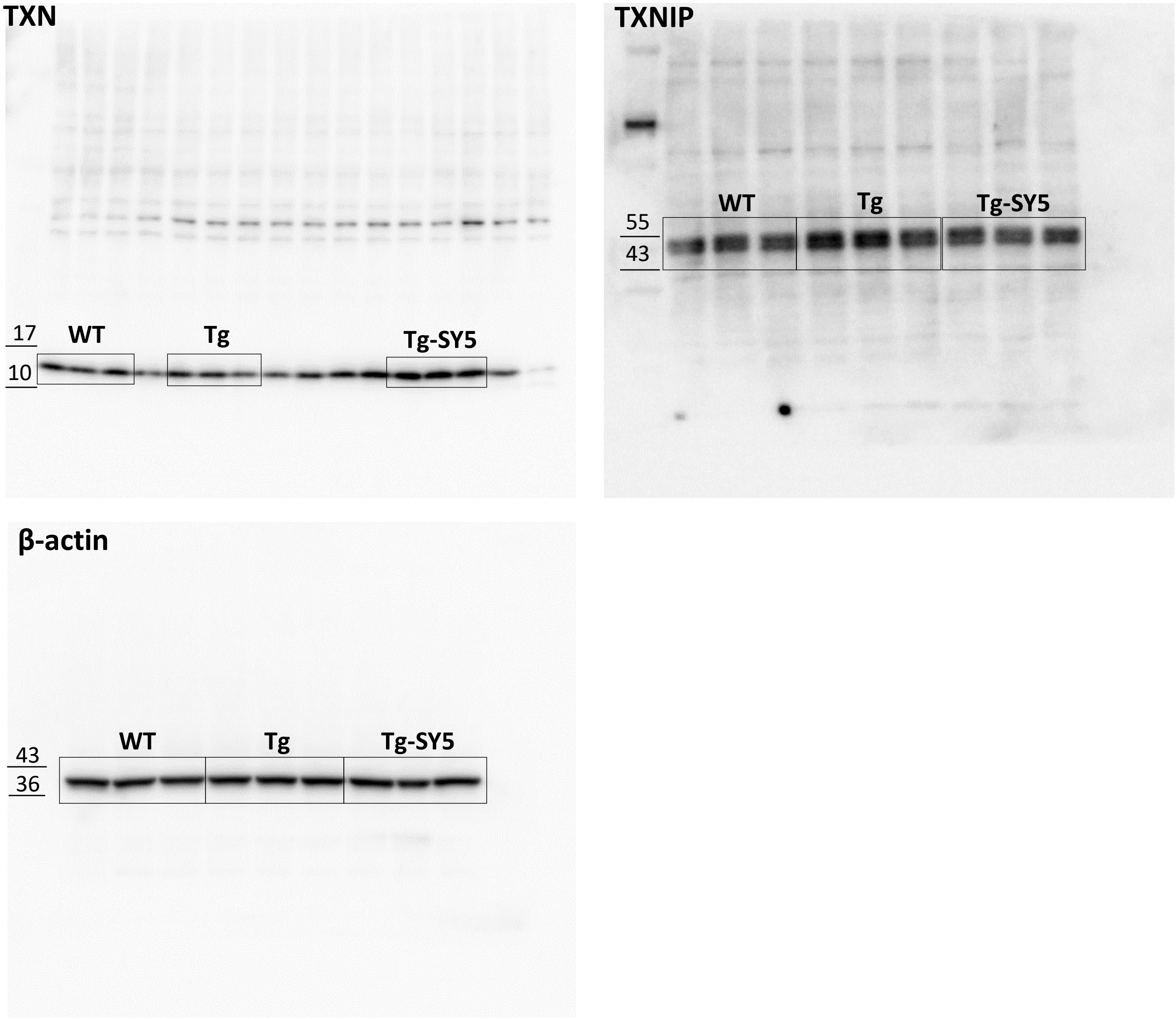

Supplementary Figure S1. Full-length blots in Figure 2a.

Supplementary Figure S2

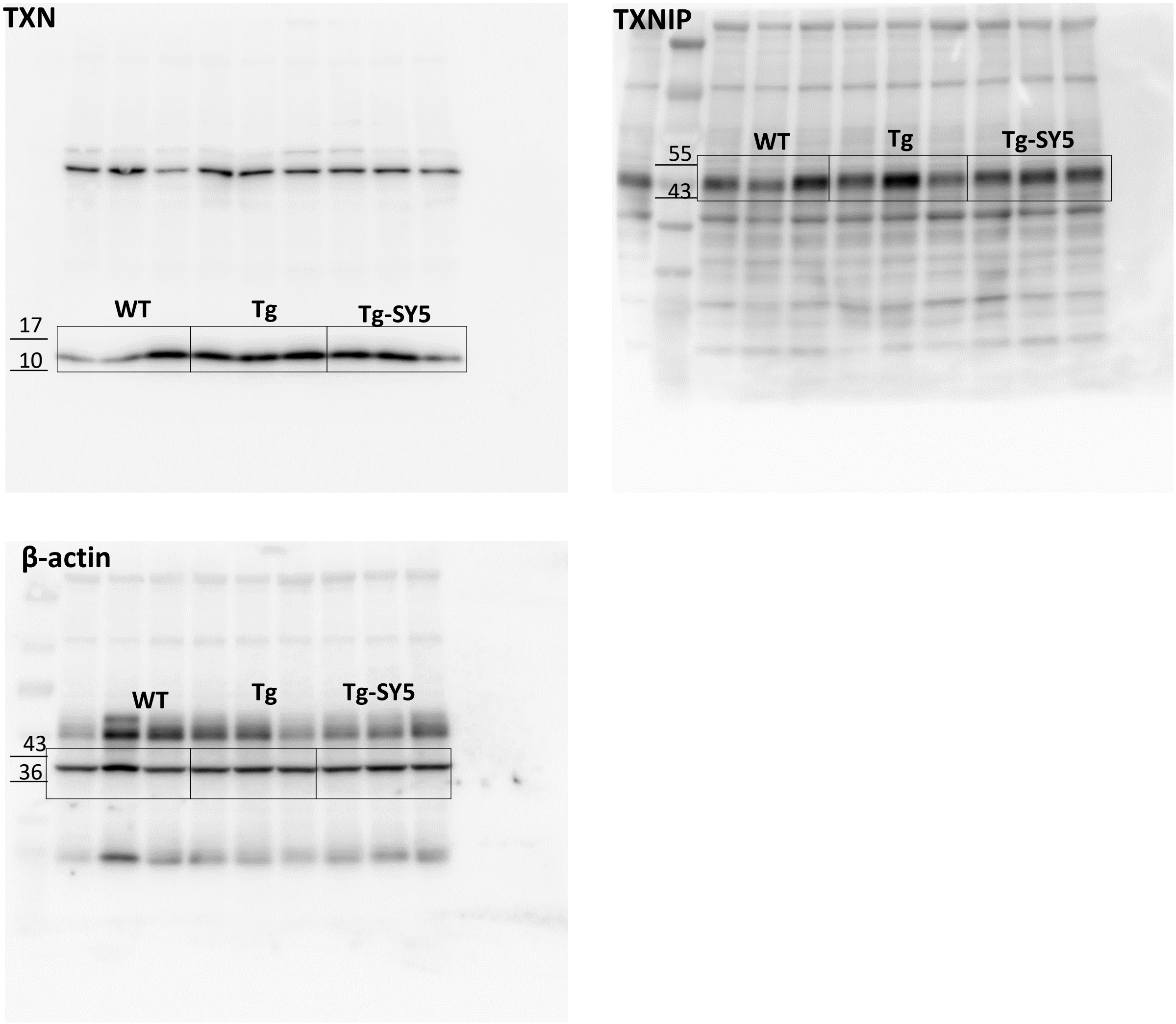

Supplementary Figure S2. Full-length blots in Figure 2b.

Supplementary Figure S3

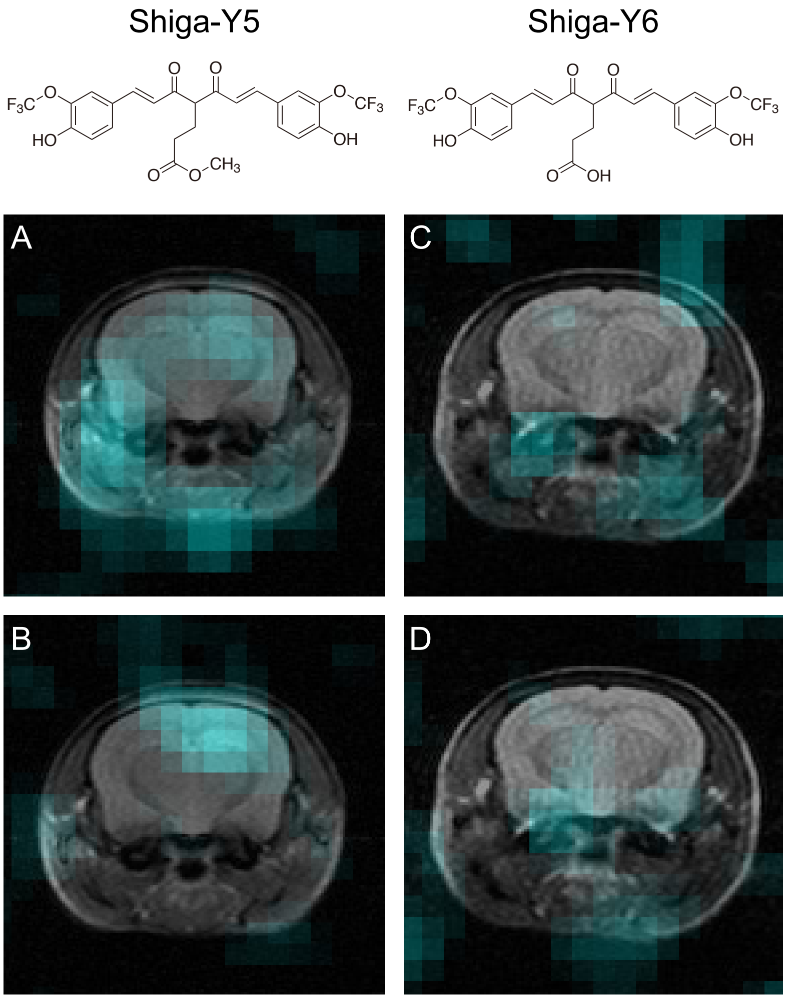

Supplementary Figure S3. ^{19}F MRI using Shiga-Y5 and Shiga-Y6. Compounds were dissolved in 20% Cremophor EL in saline at 8 mg/mL. Under anesthesia with sodium pentobarbital, each compound was injected into tail veins of 17-month-old APP transgenic mice (Tg2576 mice) at a dose of 50 mg/kg. Under continuous anesthesia with pentobarbital, MR images were taken by a 7.0 T horizontal-bore MR scanner with a volume coil tuned to both the ^{1}H and ^{19}F frequencies (300 MHz and 282 MHz, respectively). ^{1}H gradient-echo MR images of the mouse brain were obtained using a 150-ms repetition time (TR), 3-ms echo time (TE), 60° flip angle, 1.5-mm slice thickness, 24 mm × 24 mm field of view (FOV), and 256 × 128 resolution. The ^{19}F chemical shift imaging (CSI) data were collected with a 40,000 Hz spectral width, 24 mm × 24 mm FOV in the axial plane, and 56 acquisitions for each 8 × 8 phase-encoding step. A slice-selective pulse was not used, but slice selection was achieved by the sensitivity of the RF coil. Whole signals covered by coil sensitivity were acquired. (A–D) Merged images of ^{1}H MR images and ^{19}F MR images (shown in cyan). (A, B) ^{19}F MR images obtained for 1 h from 1 h after the injection with Shiga-Y5 showed ^{19}F MR signals in the brain (A). ^{19}F MR images obtained for 3 h from 3 to 6 h after the injection with Shiga-Y5 clearly showed the accumulation of ^{19}F MR signals of Shiga-Y5 in the brain, suggesting that Shiga-Y5 detected amyloid-β accumulation in the Tg2576 mouse brain. (C, D) ^{19}F MR images obtained for 1 h from 1 h after the injection with Shiga-Y6 showed no ^{19}F MR signals in the brain because of poor permeability of the blood-brain barrier (C). ^{19}F MR signals of Shiga-Y6 were not detectable in the brain even when the image was obtained for 3 h from 1 to 4 h after the injection with Shiga-Y6 (D).
